# Novel* TRAPPC11* Mutations in a Chinese Pedigree of Limb Girdle Muscular Dystrophy

**DOI:** 10.1155/2018/8090797

**Published:** 2018-07-16

**Authors:** Xike Wang, Yue Wu, Yuxia Cui, Nan Wang, Lasse Folkersen, Yuchuan Wang

**Affiliations:** ^1^Department of Pediatrics, Guizhou Provincial People's Hospital, Guiyang, Guizhou 559992, China; ^2^Sankt Hans Hospital, Capital Region Denmark, Roskilde 4000, Denmark

## Abstract

Limb girdle muscular dystrophies (LGMDs) are a heterogeneous group of genetic myopathies leading primarily to proximal muscle weakness. It is caused by mutations at over 50 known genetic loci typically from mutations in genes encoding constituents of the sarcolemmal dystrophin complex or related functions. Herein we describe the case of two siblings with LGMD that were investigated using whole-exome sequencing followed by Sanger sequencing validation of a specific double-mutation in the* TRAPPC11* gene. Further, from parental sequencing we determined the mode of transmission, a double heterozygous mutation at the maternal and paternal alleles. The two mutations detected have not been described in other patients.

## 1. Introduction

More than 50 mutations have been described for LGMD, all having either autosomal-dominant (termed LGMD1) or autosomal-recessive inheritance (termed LGMD2) [1–4]. As such, LGMD constitutes a heterogeneous group of myopathies, but all leading to proximal muscle weakness, with relative sparing of heart and bulbar muscles. The major forms of LGMD result from mutations in genes encoding constituents of the sarcolemmal dystrophin complex, e.g., laminin (*LGMD1B*), sarcoglycan (*LGMD2C-F*), and dysferlin (*LGMD2B*). Other forms have also been described, typically resulting from mutations in genes affecting muscle function involving membrane trafficking [5], muscle remodeling [6], and posttranslational modification of sarcolemmal proteins [7]. The age of onset, severity, and rate of progression vary considerably between LGMD subtypes, ranging from early childhood myopathy to adult onset with long-time preserved ambulation. Here, we reported the clinical and molecular phenotype of an autosomal-recessive form of LGMD caused by two novel* TRAPPC11* mutations in a Chinese family of Buyi origin with two affected members.

## 2. Case Presentation

The first affected individual was from a Chinese family of Buyi origin, a girl born to healthy unrelated parents after an uneventful pregnancy. The affected individual presented at age 5 with a progressive proximal muscle weakness and difficulty in standing up from sitting and walking, particularly stair-climbing. This started at approximately age 2 and gradually worsened. Physical examination was unremarkable except for mild short stature. No other muscle groups, including upper limbs, were not affected. No cataracts were observed. Echocardiogram and abdominal ultrasound scan was likewise unremarkable.

The second affected individual was a younger boy, born to the same parents. He presented at the same time, at age 3, with a similarly progressive proximal muscle weakness and the same developmental history as the first individual. Physical examination was likewise unremarkable, with no other muscle groups, affected, no remarks in ultrasound, and no cataracts.

In both individuals, there were no significant signs of extramuscular involvement. Biochemistry analysis revealed more than 100-fold increase of serum creatine kinase (CK) levels, markedly elevated serum lactate dehydrogenase level (1747 U/L) and *α*-Hydroxybutyrate dehydrogenase (1237 U/L) and mildly elevated serum glutamic-oxaloacetic transaminase (142U/L). The electromyogram (EMG) examination showed a typical muscle-derived damage in both affected children. No MRI or muscle biopsies were performed.

### 2.1. Whole-Exome Sequencing

Genomic DNA samples were extracted with Gentra Puregene Kit (Qiagen, Germany) from peripheral blood collected from the patient and his parent. The quality and quantity of genomic DNA sample were determined using a spectrophotometer (NanoDrop, USA). Genomic DNA library was prepared with Agilent SureSelect Human All Exon Kit v5 reagents, as instructed by the manufacturer's standard protocol. The enriched DNA samples were sequenced with Hiseq2000 instrument (Illumina) using 2×100 paired-end sequencing. The Illumina Sequencing Control Software (SCS) v2.8, the Illumina Off-Line Basecaller Software (OLB) v1.8, and the Illumina Consensus Assessment of Sequence And VAriation (CASAVA) v1.8 were used to produce 100 bp sequence reads. The study was approved by the Ethics Committee of People's Hospital of Guizhou Province, and written informed consent was obtained from their parents of the two affected individuals.

### 2.2. Alignment, Variant Calling, and Annotation

Burrows-Wheeler Aligner (BWA) [8] was used to align sequence reads to the human reference genome (hg19) with default parameters and variants were called using the Genome Analysis Toolkit (GATK) software package VarScan [9–11]. Coverage was determined using the CalculateHsMetrics mode of Picard software. The following analytical steps were performed only with reads that matched exonic regions including exon-intron-boundaries. SNP and insertion/deletion (indels) analysis was done by different filtering steps. The resulting list of variants was annotated with Annovar [12] that summarizes and utilizes information from external databases to assess implications and consequences of a given sequence alteration, such as amino acid change, location within a canonical splice site, and information from dbSNP along with the SNP frequency if available. Finally, a manual filtering step was carried out to prioritize relevant genes in the 30 major LGMD genes for* LGMD1B*,* LGMD2C*-*F*, and* LGMD2B*.

### 2.3. Variants Filtering

The variant detection frequency was set with a threshold for variant consideration at a minimum of 20% of the reads and an absolute read minimum coverage of 10 reads. In each case all variants listed in the most recent version of the NCBI (National Center for Biotechnology Information) dbSNP database were excluded as well as silent mutations. Low frequency frameshift and truncating mutations in any LGMD gene were considered pathogenic. Unreported nonsynonymous amino acid variants were analyzed by MutationTaster (http://www.mutationtaster.org), Polyphen-2 (http://genetics.bwh.harvard.edu/pph2), and SIFT (http://sift.jcvi.org) to assess any potentially damaging effect. Variants passing these filtering steps were considered to be most likely disease-causing and forwarded to validation process by Sanger sequencing. Additionally, genes with at least two heterozygous changes in the DNA sequence were considered to be most likely disease-causing, even though homozygous variants were not completely withdrawn.

### 2.4. Sanger Sequencing

We validated the candidate variations by Sanger sequencing in the two affected individuals and their parents. PCR primers were designed with Primer3 tool (http://frodo.wi.mit.edu/primer3/) to contain the mutation sites and their flanking regions (PCR primers and PCR reaction conditions are available upon request). PCR amplifications were inspected for single band of expected sizes on agarose gels before purification with Agencourt AMPure on Biomek NX (Beckman Coulter, USA). Sequencing was achieved using the automated ABI Prism 3730xlDNA Sequencer in combination with the Big Dye Terminator Cycle Sequencing Ready Reaction Kit 3.1 (Applied Biosystems, USA), and purification of sequencing reaction was performed with Agencourt CleanSEQ on Biomek NX(Beckman Coulter, USA). Sequences were assembled and analyzed with Mutation Surveyor software (SoftGenetics, USA).

## 3. Results

**Figure 1:**
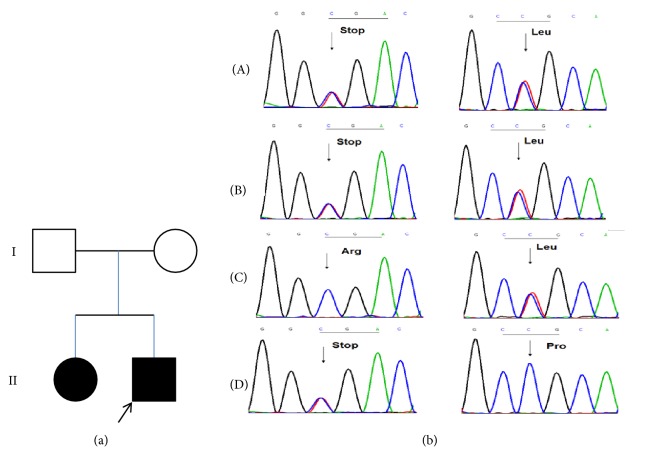
(a) Pedigree of family and (b) Sanger sequencing results from TRAPPC11 mutations. The left column shows the c.1192C>T (p.Arg398*∗*) mutation and the right column shows the c.3014C>T (p.Pro1005Leu) mutation. The two firsts row ((A) and (B)) shows results from the children. The next rows ((C) and (D)) show the results from the parents.

**Figure 2:**
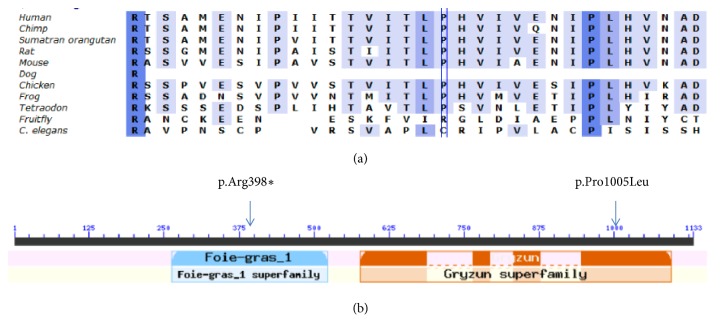
(a) Alignment of the c.1192C>T (p.Arg398*∗*) mutation across species, illustrating a conserved region. (b) Indication of both c.1192C>T (p.Arg398*∗*) and c.3014C>T (p.Pro1005Leu) mutations and their position in relation to functional domains of the gene.

Exon-wide sequencing revealed two heterozygous mutations in exon 11 and exon 27, respectively, of* TRAPPC11* in both affected children. Both of them were single base substitution c.1192C>T (p.Arg398*∗*) and c.3014C>T (p.Pro1005Leu). Sanger sequencing confirmed the two heterozygous mutations in the patients and each was inherited from one of the parents (c.1192C>T (p.Arg398*∗*) from the mother and c.3014C>T (p.Pro1005Leu) from the father (Figure 1). The two mutations were found to be conserved across multiple species and in known functional domains of the* TRAPPC11* gene (Figure 2).

So far, there were only four mutations in* TRAPPC11* reported in patients. The two mutations detected in our patients have not been described in patients. The c.1192C>T (p.Arg398*∗*) was observed as rs140403642 in the NHLBI GO Exome Sequencing Project database (ESP, http://evs.gs.washington.edu/EVS/) with a MAF of 0.000077, c.3014C>T (p.Pro1005Leu). The variants were found as in the ExAC database in 1 and 5 samples, corresponding to a frequency of 8.2e-06 and 4.1e-05, respectively. In ExAC they were not detected in non-Caucasian ethnicity samples and thus represent novel mutations in Asian ethnicity. Likewise, they were not found in the 1000 genomes database and they were undescribed in the ClinVar database.

## 4. Discussion

In this report, we identified by whole-exome sequencing a compound heterozygous mutations in* TRAPPC11 *(AK022778.1), i.e., c.1192C>T (p.Arg398*∗*) and c.3014C>T (p.Pro1005Leu), in two affected members of a Chinese family with LGMD. Previous findings include the whole-exome sequencing combined with linkage analysis of a Syrian family with limb girdle muscular dystrophy type 2S (LGMD2S;OMIM#615356). Further, Bogershausen et al. identified a homozygous mutation in the* TRAPPC11* gene (G980R) [13]. The authors also detected a different homozygous mutation in the* TRAPPC11* gene (Ala372_Ser429del) in affected members of 2 Hutterite families with a slightly different phenotype. The G980R mutation occurred in the gryzun domain, whereas the deletion occurred in the foie gras domain. Similarly, c.1192C>T (p.Arg398*∗*) mutation and c.3014C>T (p.Pro1005Leu) mutation reported in this study also occurred in the foie gras domain and the gryzun domain, respectively (Figure 2). Recent additional work further expands the breadth of findings, as summarized in Table 1 [14–16].

The affected individuals with* TRAPPC11* mutations were described with two groups of clinical manifestations: one with more prominent muscular and skeletal symptoms and the other with microcephaly, hyperkinetic movements, ataxia, and intellectual disability, which was discussed as a reflection of the difference of the two genotypes, Gly980Arg and Ala372_Ser429del [13]. Most recently, Liang et al. reported a Chinese girl harboring a compound heterozygous c.2938G > A/c.661-1G > T mutations in* TRAPPC11* presenting congenital muscular dystrophy, fatty liver, and infantile-onset cataract, demonstrating the broad spectrum of disease phenotypes arising from* TRAPPC11* mutation in human [17]. The affected individuals from the Chinese family of LGMD2S reported herein were mainly characterized with progressive proximal muscle weakness resulting in impaired ambulation, difficulty to climb stairs, and increased serum creatine kinase. We did not observe noticeable features of nervous and hepatic involvement in the two patients with LGMD2S. The results clearly revealed that there is significant variability in phenotypes of* TRAPPC11* mutations.

*TRAPPC11* is a component of the TRAPP multisubunit tethering complex involved in intracellular vesicle trafficking [18]. Patient cells from both groups showed increased fragmentation of the Golgi apparatus and decreased amounts of the mutant proteins. Studies in yeast suggested that the mutant missense protein lost the ability to interact properly with other TRAPP proteins. Patient cells also showed altered protein transport along the secretory pathway, with a delayed exit from the Golgi and a defect in the formation and/or movement of late endosomes/lysosomes [13]. Liang et al. did not observe full-length* TRAPPC11* protein in the patient harboring c.2938G > A/c.661-1G > T compound heterozygous mutations [17]. The findings suggested that altered membrane trafficking is the underlying molecular mechanism of this disease spectrum. Interestingly* TRAPPC11* has also been associated with glycosylation [19] and later studies on zebra fish have confirmed that* TRAPPC11* is involved in protein glycosylation [20], providing for a more detailed hypothesis on disease mechanism in this case report.

In conclusion, this study widens the phenotype of* TRAPPC11* mutation related disorder and provides a conclusive case report for LGMD.

## Figures and Tables

**Table 1:** Comparison of the present patient and previously reported patients with *TRAPPC11 *mutations. NR: not reported.

	**c.2938G > A homo**	**c.1287 + 5G > A homo**	**c.2938G > A, c.661-1G > T**	**c.1893+3A>G, g.4:184,607,904A>G**	**c.142C>T, g.27324T>A **	**c2330A>C, c513-516 (delTTTG)**	**c. 1192C>T, c.3014C>T**
Number of patients	3	5	1	4	1	2	2
Age of onset of muscle symptoms	Early school age	Early childhood onset	Around 1-year-old or even earlier	NR	NR	Early childhood onset	Approx. 2-year-old
Muscle pathology	Proximal weakness, myalgia, cramps Myopathic	Mild weakness and hypotonia, myopathic	Proximal weakness, hypotonia Dystrophic	Weakness, Dystrophy, Atrophy	Hypotonia	Mild weakness and hypotonia, myopathic	Proximal weakness
CK (IU/L)	600~2800	300~1000	6000~9000	-	NR	800~7000	7989
Head circumference	Within normal limits	<3rd percentile	(−)	(-)	NR	<1st percentile	Within normal range
Intellectual disability	(−)	(+)	Borderline	+	(+)	(+)	(-)
Ataxia	(−)	(+)	(−)	(-)	(-)	(-)	(-)
Choreiform movement	(−)	(+)	(−)	(-)	(-)	(-)	(-)
Other neurological problems	(−)	Generalized seizure Abnormal EEG	(−)	(-)	(-)	Generalized seizure, Abnormal EEG	(−)
Neuroimaging Cardiac involvement	Enlarged right ventricle	Mild cerebral atrophy	Reduced white matter volume	Cerebral atrophy	NR	Brachycephaly	NA
Skeletal involvement	Hip dysplasia, scoliosis	Limb asymmetry	Lordosis	(-)	Scoliosis, skeletal anomalies	(-)	(-)
Ocular involvement	Esotropia and myopia, cataract	Exophoria, anisometropia, and amblyopia	Infantile—onset cataract	(-)	NR	Cataracts	(-)
Hepatic involvement	(−)	(−)	Steatosis	(-)	NR	(-)	GOT:142U/L
Reference	(13)	(13)	(14)	(15)	(16)	(17)	Current study
